# Change detection and repetition detection reflect functionally distinct forms of visual working memory

**DOI:** 10.3758/s13421-025-01749-2

**Published:** 2025-06-24

**Authors:** Stephanie Norris, Andrew P. Yonelinas

**Affiliations:** 1 Department of Psychology, University of California, Davis, CA, USA

**Keywords:** Visual working memory, Change detection, Repetition detection, Recollection, Receiver operating characteristics

## Abstract

To examine the roles of change detection and repetition detection in visual working memory, we analyzed three working memory tests expected to rely differentially on these processes. Subjects studied an array of colored squares and then completed three tests. In the *complex-probe* test, subjects indicated whether a test array matched the study array or if an item’s color changed. In the s*ingle-probe* test, they judged whether a single item’s color matched the study color, and in the *item recognition* test, they identified whether a centrally presented color was studied. We collected same/different confidence responses and analyzed receiver operating characteristics (ROCs) to evaluate memory strength distributions for changed and repeated trials, using a mixture signal detection model to estimate each process. As expected, the *complex-probe* test showed more high-confidence memory for changed trials, while the *item recognition* test showed more high-confidence memory for repetitions. The *single-probe* test showed similar or lower-confidence memory for both trials. Moreover, model estimates indicated that the probability of recollecting a change was higher in the *complex-probe* than in the *item recognition* tests, and the probability of recollecting a repetition was higher in the *item recognition* than the *complex-probe* tests. The s*ingle-probe* test showed moderate recollection for both. These results show that change detection and repetition detection are functionally dissociable, with test-type affecting their contributions to working memory. These findings have implications for studying populations, such as aging, that may exhibit impairments in one or the other and raise the question of whether different neural systems underlie these processes.

## Introduction

The ability to detect changes in our visual environment, as well as the ability to detect repetitions, are two core functions of visual working memory (VWM), and together they enable us to successfully navigate and interpret our complex visual surroundings. For example, while driving along a road, we may suddenly notice the appearance of an object (i.e., change detection), prompting us to brake to avoid hitting a pedestrian. However, if the object were to remain stationary (i.e., repetition detection), this might indicate that it is safe to continue driving. Recent work has suggested that the extent to which subjects utilize change detection and repetition detection may vary across different types of working memory tests. That is, in some cases, the mnemonic information carried by changed trials appears to be stronger than that carried by repeated trials, whereas in other cases, the reverse appears to be true (Yonelinas, 2024). However, these observations are based on comparing results from across different experiments that varied not only in the types of memory tests used but also in stimulus type and specific test procedures. Thus, it is not yet known whether the type of memory test determines the extent to which working memory relies on change detection and repetition detection, or whether some other factor was responsible for the observed differences.

## The role of change detection and repetition detection in different memory tests

**Fig. 1:**
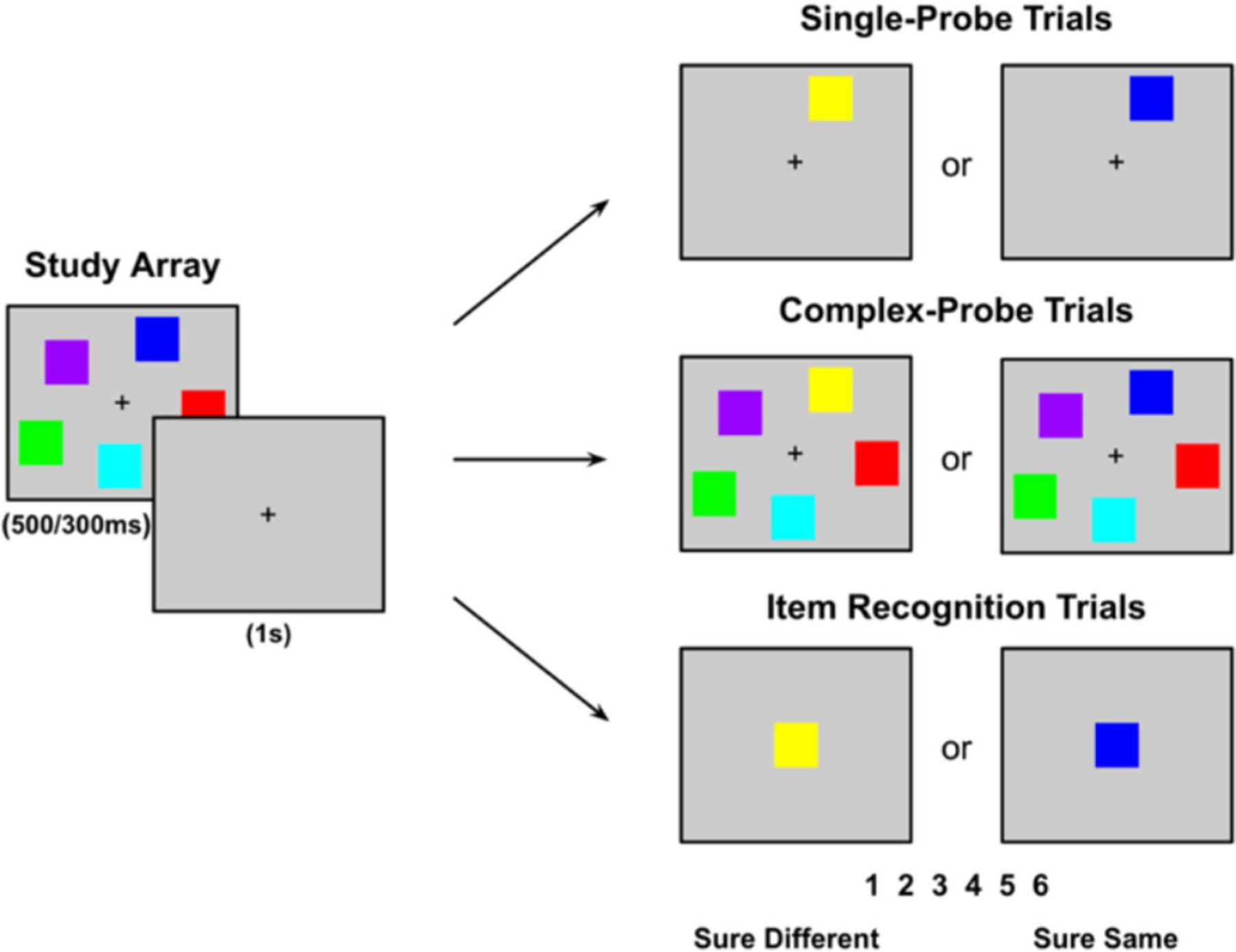
Three common types of working memory tests: *single-probe*, *complex-probe*, and *item recognition*. In Experiment 1 in the current study, participants viewed a study array for 500 ms and, after a 1-s delay were presented with a test stimulus that was either the same as or different from the study array. They indicated whether the item was the “same” or “different” using a 6-point confidence scale. The three test types (i.e., *single-probe*, *complex-probe,* and *item recognition*) were presented in random order. In Experiment 2, the study array duration was reduced to 300 ms, and the test types were presented in a blocked format rather than randomized

The extent to which subjects make use of change detection or repetition detection in working memory is largely unexplored. However, if subjects can maintain qualitative information about the study event, then the extent to which that information supports the detection of changes versus the detection of repetitions should depend on the type of memory test (Aly & Yonelinas, 2012; Rhodes et al., 2018; Rouder et al., 2008, 2011; Yonelinas, 2024). For example, consider a *single-probe* test (see upper-right panel of Fig. 1) in which subjects must indicate whether a studied item was presented in a given color. In this test, if a subject can maintain the color of the probed item, then this information should be useful in accepting an old item as old (i.e., “It was blue”) and in rejecting a new item as having changed (i.e., “It was blue, not yellow”). So, in this case, recollection is expected to contribute similarly to the detection of both changes and repetitions.

In contrast, in a *complex-probe* test—also known as a “whole-array” or “whole-display” test (see middle-right panel of Fig. 1)—in which subjects are presented with an entire test array and asked to indicate if the probe image is different in any way from the study array, recollection is no longer equally useful in detecting repetitions and in detecting changes. That is, if a subject can maintain any one of the studied items that changed, this is sufficient to reject the test array as new (i.e., change detection). However, determining that the test array is the same as the study array (i.e., repetition detection) requires maintaining more than a single item or feature. That is, to be certain that an array is repeated requires memory for the entire study array. In this way, recollection will be more useful in detecting changes than in detecting repetitions.

Finally, as illustrated in the bottom-right panel of Fig. 1, in an *item recognition* test, in which subjects are presented with a test probe and must indicate if the probe color was studied, memory for repetitions should now be more useful than memory for changes. That is, in this type of test, if the subject can remember that the color of a study item matches the test probe, then this should be sufficient to accept that the probe color was studied. However, rejecting a probe color as having not been studied requires maintaining more than just the color of any single studied item. That is, to be certain that an item is new requires remembering the color of all the studied items.

## Prior visual working memory ROC results

**Fig. 2:**
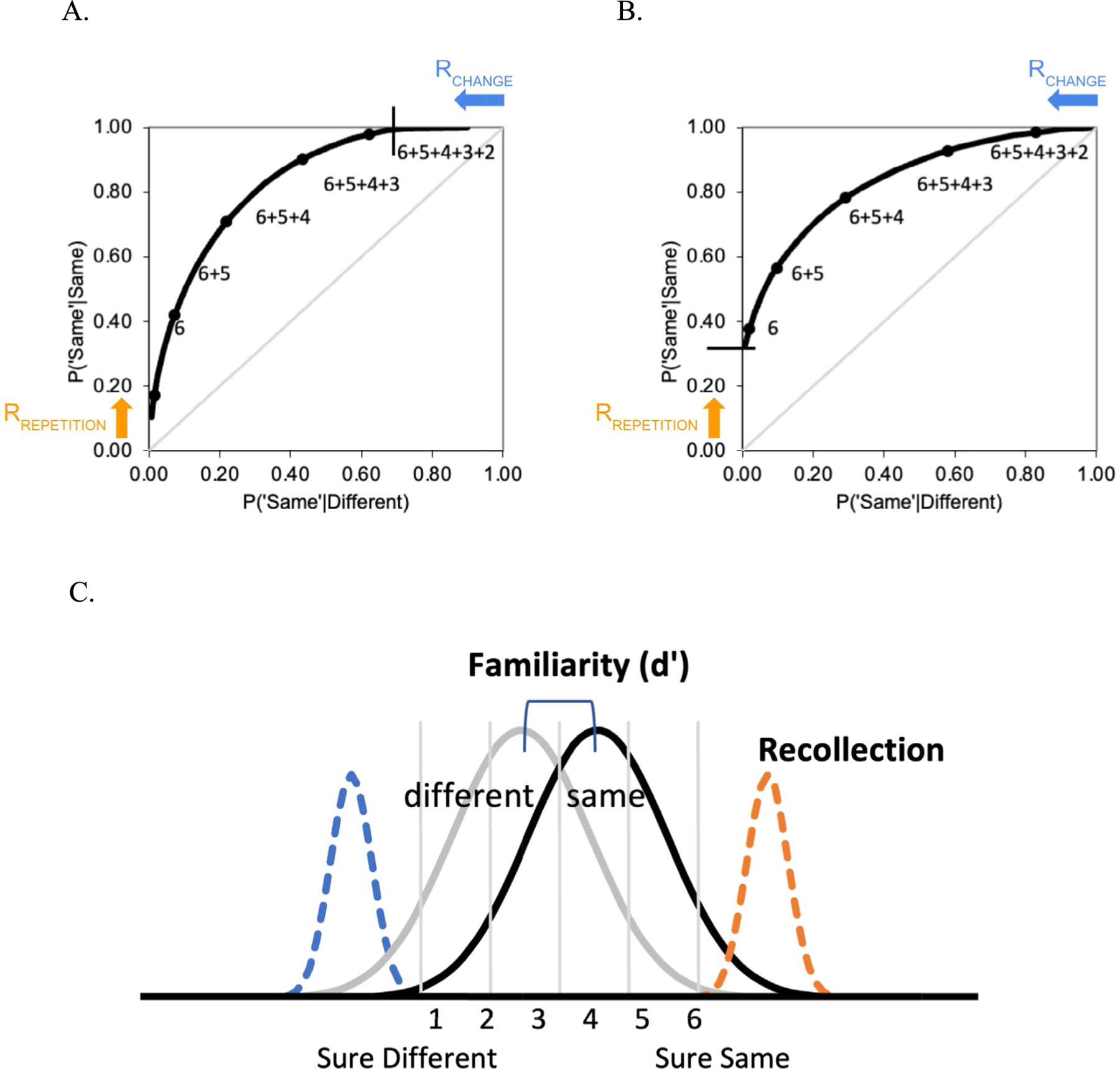
**A**. An ROC with a steep slope, fit to the mixture model. **B.** An ROC with a shallow slope, fit to the mixture model. **C.** The memory strength distributions for familiarity and recollection in the mixture model

A recent review of VWM ROC studies (Yonelinas, 2024) noted that the underlying memory strength distributions for changed and same trials across the three test types appeared to be at least broadly consistent with the expectations described above. In these studies, subjects first encoded a set of items and, following a brief delay, were presented with a test probe and required to indicate whether it was the “same” or “different” using a confidence rating scale (e.g., 1 = *sure different* to 6 = *sure same*). Performance in these studies was examined by plotting the hit rate (i.e., the probability of accepting a repeated item as having been studied) against the false-alarm rate (i.e., the probability of incorrectly accepting a changed item as having been studied), across levels of response confidence. This resulted in receiver operating characteristics (ROCs) like those illustrated in Fig. 2 A–B. For example, the leftmost point reflects the proportion of the “same” and “different” trials receiving a high confidence “same” response (i.e., all the 6 responses), and the next point includes the next most confident responses (i.e., the 6s and 5s). In this way, a 6-point confidence scale produces five ROC points, with the last point not being plotted as it is constrained to be 1,1.

The confidence ROCs reported in VWM studies are typically curved downward, but there is considerable variability in the observed shape of the functions with respect to how asymmetrical they are. For example, the ROC in Fig. 2A is asymmetrical and is pushed to the left such that it appears to intersect the upper *y*-axis. The asymmetry can be measured by plotting the ROC in *z*-space and measuring the slope of the function (Swets, 1961).

A perfectly symmetrical ROC will have a *z*-slope of 1.0, whereas the ROC that is pushed to the left will have a *z*-slope of greater than 1.0, thus we will refer to this as a curvilinear ROC with a steep slope. Conversely, an ROC that is asymmetrical in the opposite direction, as illustrated in Fig. 2B (i.e., the ROC appears to be pushed up on the left side), will have a *z*-slope of less than 1.0, and so is a curvilinear ROC with a shallow slope.

In *complex-probe* tests, confidence ROCs have been examined across several experiments and are generally found to be asymmetrical with a steep slope, as in Fig. 2A (e.g., Aly & Yonelinas, 2012; Goodrich et al., 2019; Wilken & Ma, 2004). For example, in a series of experiments examining confidence rating *complex-probe* tests, the resulting ROCs for sets of objects, as well as for scenes, faces, and fractals, were all asymmetrical with a steep slope (Aly & Yonelinas, 2012). Another study examined working memory for complex Gabor patches in which either the color, orientation, or location of the stimulus could change, and also reported asymmetrical ROCs with a steep slope (Goodrich et al., 2019). Similarly, in a series of experiments examining confidence ratings for colored squares, as well as the orientation and spatial frequency of Gabor patches across various set sizes, the reported ROCs were curvilinear and asymmetrical, with a steep slope (Wilken & Ma, 2004). Note that in some previous studies (e.g., Wilken & Ma, 2004), the *x*- and *y*-axis in the reported ROC figures were reversed, which inverted the resulting ROC, but this does not impact the theoretical interpretation of the results.

In contrast, in experiments examining *item recognition*, the resulting ROCs are generally asymmetrical with a shallow slope, as in Fig. 2B. For example, across three experiments examining *item recognition* for a rapidly presented series of objects, the ROCs were found to be curved and asymmetrical, with a shallow slope rather than a steep slope (Robinson et al., 2020). Shallow slopes have also been reported in experiments examining *item recognition* for letters that were presented simultaneously (Yonelinas & Jacoby, 1997, as cited in Yonelinas, 2024). The finding that *item recognition* in VWM produces ROCs with a shallow slope is also consistent with results from *item recognition* tests of long-term episodic memory, where the observed ROCs almost always have a shallow slope (for a review, see Yonelinas & Parks, 2007). However, Yotsumoto et al. (2008) reported *z*-slopes greater than 1.0 in an *item recognition* test for Gabor patches. We consider this study in more detail in the discussion.

Finally, in studies examining *single-probe* recognition, the resulting ROCs are curved downward, and they are most often symmetrical, such that the resulting slope of this function is close to 1.0. For example, symmetrical curvilinear confidence ROCs were observed for *single-probe* tests of colored squares in two experiments examining the effects of different types of visual masks (Xie & Zhang, 2017b). In addition, symmetrical curvilinear ROCs were observed in *single-probe* confidence ROCs for three experiments examining memory for line orientations (Xie & Zhang, 2017a). However, note that a fourth experiment in that study did produce noticeably asymmetrical ROCs with a steep slope (i.e., when line segments were joined with a faint line to form overall configurations). Finally, in a series of three confidence rating ROC experiments for *single-probe* color memory, symmetrical ROCs were also observed (Robinson et al., 2020).

In sum, in *complex-probe* tests of VWM, the confidence ROCs from many studies using a variety of different materials exhibit a steep slope. In contrast, in *item recognition*, the ROCs are generally found to exhibit a shallow slope, and in *single-probe* tests, the ROCs are generally found to be symmetrical.

## How do ROCs relate to change detection and repetition detection?

Yonelinas (2024) interpreted the existing ROC results in the context of a mixture signal detection model (or the dual-process signal detection model), which suggested that the three tests relied differentially on change detection and repetition detection. Other models will be considered further in the discussion section. The mixture model (see Fig. 2C) assumes that working memory performance reflects a mixture of familiarity- and recollection-based responses (for applications of the model to working memory, see Aly & Yonelinas, 2012; Goodrich et al., 2019; Xie & Zhang, 2017a, 2017b; for applications to episodic memory, see Yonelinas, 1994; Yonelinas & Parks 2007). In working memory, the model assumes familiarity reflects the extent to which the test item is perceived as familiar, whereas recollection reflects the ability to actively maintain qualitative information about a study item. That is, it is assumed that repeated items will be perceived as more familiar than changed items because repeated items will more strongly match the contents of memory. In addition, familiarity is assumed to vary from item to item such that the familiarity distributions for the “same” and “different” items will be normally distributed and overlapping, as in classic signal detection theory (i.e., the gray distributions in Fig. 2C). This Gaussian memory strength signal is broadly consistent with distributed cortical neural network models relying on Hebbian learning (e.g., Elfman et al., 2014; Norman and O’Reilly, 2003). Thus, familiarity will produce an ROC that is curvilinear and symmetrical, and so would produce a *z-*slope of 1.0. Familiarity strength reflects the distance between the two distributions (*d′*), such that increases in familiarity lead the ROC to be more curvilinear and pushed away from the chance diagonal.

However, in addition to assessing familiarity, it is assumed that subjects can also recollect qualitative information about the study array (e.g., they can actively maintain that a specific study item was blue). Recollection is assumed to be thresholded, in the sense that it can sometimes fail (e.g., subjects may have failed to attend to a study item, it was not actively maintained during the retention interval, or there was a retrieval failure). Recollection is assumed to rely on frontoparietal attention networks that result in the active maintenance of attended items (Yonelinas et al., 2024). Most importantly, because recollection carries qualitative information about the attended items, it can impact performance in two different ways. First, it should support the *detection of changes*, or what is sometimes referred to as the recollection of newness. That is, if a subject is actively maintaining that an item is blue, and that item then changes to yellow, this should lead to a highly confident “sure different” response. This is illustrated as the dashed blue distribution on the left side of Fig. 2C and would result in an ROC that is no longer symmetrical but rather would exhibit a steep slope as in Fig. 2A. The probability of detecting a change will be estimated by the upper intercept of the resulting ROC, which will move further to the left as change detection increases. In contrast, however, recollection should also support the *detection of repetitions*, or recollection of oldness. That is, if a subject is actively maintaining that an item is blue, and that item is then repeated, this should lead to a highly confident “sure same” response. This is illustrated as the dashed orange distribution on the right side of Fig. 2C and would result in an ROC with a shallow slope, as in Fig. 2B. In this case, recollection of repetition would be estimated by the left intercept of the ROC.

According to the mixture model, then, the steep ROC slopes observed in *complex-probe* tests arise because these tests rely more heavily on the recollection of changes than on the recollection of repetitions, whereas the shallow ROC slopes observed in *item recognition* tests reflect a greater reliance on the recollection of repetitions. Moreover, the symmetrical ROCs often observed in *single-probe* tests presumably arise because of a roughly equal contribution of both of these processes.

## The current experiments

The current experiments were designed to overcome several perceived limitations of previous studies. The results described above are consistent with the claim that ROC shape differs systematically across different types of working memory tests. However, these prior studies differed in a variety of ways other than just the type of test they used. For example, although the *complex-probe* ROC results have been observed in many studies using a wide variety of materials, *item recognition* ROCs have been examined for objects studied sequentially or for sets of letters that may have promoted sequential phonological rehearsal. Thus, it is not clear whether the differences in ROC shape are due to the test type itself, the materials used, or the study presentation format. In addition, these observations were based on an examination of the average ROCs reported in those earlier studies. Although average ROCs are generally found to be reflective of a majority of individual subjects, averaging artifacts can impact the shape of nonlinear functions like ROCs (Yonelinas & Parks, 2007). Finally, the existing studies do not allow us to determine if the observed differences in ROC shape reflect differences in the working memory processes that subjects employ in different types of tests or differences in the type of encoding strategies that subjects adopted. That is, in all of the published work, subjects were repeatedly tested on the same test procedure, so they could have adopted different encoding strategies in the different tests. For example, in *complex-probe* tests, subjects may focus on encoding color-location information, whereas in *item recognition* tests, they may focus more exclusively on color information because the location information is not relevant.

To directly test whether change and repetition detection differ systematically across test types, we contrasted three common working memory tests while holding the materials and subjects constant. In addition, to determine whether the processes that subjects use depend on whether they can adopt task-specific encoding strategies, we examined performance when test type varied randomly from trial to trial (Experiment 1) and when the different tests were conducted in separate blocks (Experiment 2).

## Experiment 1: Random testing

### Methods

#### Participants

Thirty-one participants were recruited from the undergraduate population at the University of California, Davis, and took part in the study for course credit. All had normal or corrected-to-normal visual acuity and normal color vision. Informed consent was obtained prior to participation. Eight participants were removed due to a lack of range in responses to evaluate ROC shape (i.e., having less than three ROC points), giving us a total sample of 23. Of these participants, ages ranged from 18 to 23 years.

#### Stimuli

The tests were presented on a 17-in. monitor with a resolution of 1,920 × 1,080 pixels. The software PsychoPy (Version 2023.2.3) was used for stimuli presentation and response recording. The colored squares were displayed against a neutral gray background (8-bit RGB: 128, 128, 128), and their dimensions were standardized at 50 × 50 pixels. Table 1 shows the RGB values for each of the nine possible colors.

#### Procedure

The experiment included three variations of a colored square working memory test modeled after Luck and Vogel (1997): a *single-probe* test, a *complex-probe* test, and an *item recognition* test (see Fig. 1). For each participant, testing was completed in a single 50-min session, and detailed instructions and practice trials were provided beforehand to ensure participants’ familiarity with each test before formal data collection began.

For each trial, five colors were randomly selected from the specified RGB list (Table 1) to form the study array. The locations of the squares in each trial were randomly selected but were nonoverlapping and excluded from the central location. Each trial began with a central fixation cross (+), which remained visible throughout the trial. The experiment included a randomly intermixed set of 120 trials for each of the three tests (*single-probe*, *complex-probe*, and *item recognition*), with each test consisting of 60 “same” trials and 60 “different” trials. The study array was presented for 500 ms, followed by a 1-s fixation screen, and then a test array was presented and remained onscreen until the subject responded. For *complex-probe* “same” trials, the study array was represented as the test array, maintaining all five colored squares in the same locations, whereas for “different” trials, the array was the same except that one square was randomly changed to a new color that was not present in the study array. For *single-probe* “same” trials, the test array featured only one square in its original location with the same color as in the study array, whereas for the “different” trials, the color of the single square was randomly replaced with a new color that was not part of the study array. For *item recognition* “same” trials, one square from the sample array was displayed in the center of the screen, and for “different” trials, the square was presented in the center of the screen but with a color not originally present in the study array.

For each test array, participants made a “same/different” judgment on a keyboard using a 6-point confidence scale, which was visible at the bottom of the screen throughout the response window (1 = *sure different*, 2 = *probably different*, 3 = *maybe different*, 4 = *maybe same*, 5 = *probably same*, 6 = *sure same*). After the participants’ response and a delay of 1 second, the next random trial would initiate.

#### Data analysis

All statistical analyses were performed using RStudio (Version 2025.05.0 + 496). Data processing, statistical testing, and visualization were carried out using several R packages, including *dplyr* (for data manipulation), *rstatix* (for statistical tests and ANOVA procedures), *AICcmodavg* (for model selection based on Akaike information criterion), and *ggplot2* (for data visualization).

Analyses were conducted using within-subjects (repeated-measures) designs, with both subject and test type incorporated into the statistical models. Specifically, test type was treated as a within-subjects factor, as all participants completed each condition. To account for individual variability, subject was modeled as a random factor. In the repeated-measures analysis of variance (ANOVA), variability due to individual differences (subject) and their interaction with within-subjects factors (e.g., test type) was used to partition the error term. This approach allowed for accurate estimation of within-subject effects and proper assessment of the effect of test type. In addition to the ANOVA, paired-samples *t*-tests were used to examine planned comparisons between specific conditions. Where appropriate, Bonferroni corrections were applied to control for multiple comparisons and reduce the risk of Type I errors.

Our sample sizes were guided by prior ROC studies of recognition and working memory (e.g., Aly & Yonelinas, 2012; Hawkins et al., 2025; Yonelinas, 1994; Yonelinas & Parks, 2007), which typically included 20–30 participants and demonstrated sufficient sensitivity to estimate recollection and familiarity. Following this precedent, we tested 23 participants in Experiment 1 and 29 participants in Experiment 2. We also conducted an a priori power analysis using G*Power (Faul et al., 2007), specifying a one-tailed paired-samples *t*-test, α = 0.05, and desired power = 0.80. The expected effect size was based on a previous study that found a significant difference in recollection (Pd) between discrete and global change conditions, *t*(36) = 3.15, *p* = 0.003, corresponding to a Cohen’s *d* of 0.52. The power analysis for our study indicated that a minimum of 25 participants would be required to detect an effect of this magnitude (noncentrality parameter δ = 2.60, critical *t* = 1.71). The sample size in Experiment 2 exceeded this threshold, and the sample size in Experiment 1, while slightly smaller, still fell within the range used in similar studies.

### Results

#### ROC shape

**Fig. 3:**
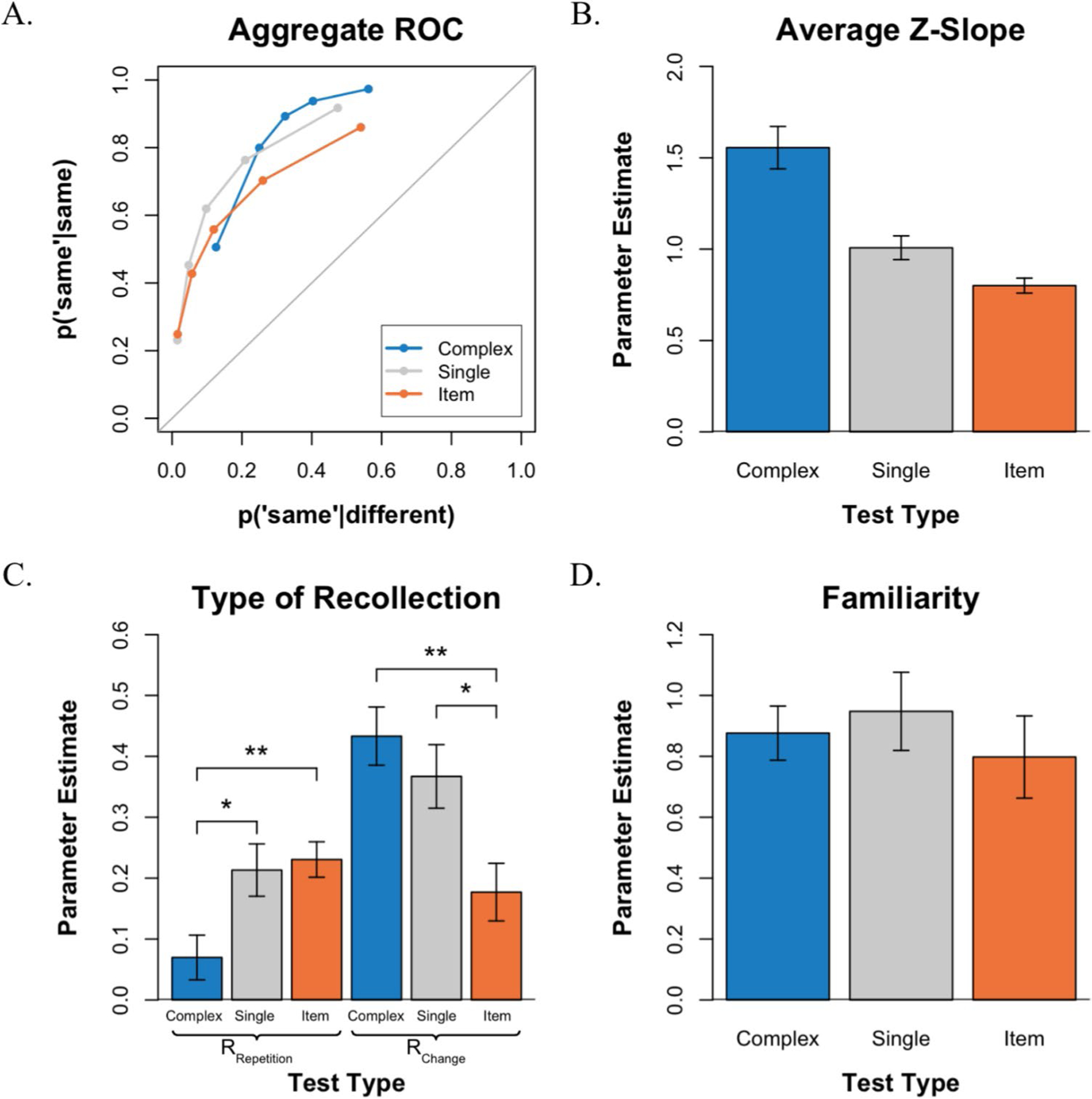
VWM performance for each test in Experiment 1. **A.** Average ROCs for the three tests. **B.** Average *z*-slope estimates across tests. **C.** Estimates of recollection type (R_Repetition_ vs. R_Change_) across tests. **D.** Estimates of familiarity across tests. *p < 0.05, **p < 0.01

Performance was measured by plotting receiver operating characteristics (see Fig. 3A). The distribution of confidence responses in each test condition can be found in Supplementary Table 1. ROCs plot the hit rate on the *y-*axis against the false-alarm rate on the *x*-axis as a function of confidence, such that the leftmost point of each ROC includes only the sure “same” responses (i.e., 6s), the next point includes the 6s and 5s, and so forth. In this way, the left side of the function is driven primarily by the sure “same” responses, whereas the right side of the function is driven primarily by the sure “different” responses. Overall, VWM discriminability was measured as the area under the curve (AUC), and the asymmetry of the functions was assessed by the slope of the function when plotted in *z*-space.

Consistent with previous ROC studies of VWM, all three ROCs are curvilinear and exhibit an inverted U-shape (see Fig. 3A). Most notably, the ROCs in the three test conditions show varying degrees of asymmetry. As expected, the *complex-probe* ROC exhibits a steep slope, the *item recognition* ROC exhibits a shallow slope, and the *single-probe* ROC appears more symmetrical. To quantify the degree of asymmetry, we plotted the average ROCs in *z*-space and examined the average ROC *z*-slopes (see Fig. 3B). An examination of that figure indicates that the slope was steepest in the *complex-probe* test, shallowest in the *item recognition* test, and very close to symmetrical in the *single-probe* test. A one-way repeated-measures ANOVA revealed a significant effect of test condition on the *z*-slope, *F*(2,44) = 26.02, *p* < 0.001. Specifically, in the *complex-probe* test, the mean *z*-slope was 1.556, which was significantly greater than 1.0, *t*(22) = 4.792, *p* < 0.001. In contrast, in the *item recognition* test, the mean *z*-slope was 0.800, which was significantly less than 1.0, *t*(22) = − 4.868, *p* < 0.001, and in the *single-probe* test, the mean *z*-slope was 1.008, which did not significantly differ from 1.0, *t*(22) = 0.116, *p* > 0.05. Finally, although overall performance across the three tests was roughly comparable, as evidenced by the overlapping ROCs, an examination of the AUC indicated that discriminability varied across tasks, *F*(2,44) = 8.01, *p* < 0.01. Further comparisons using paired *t*-tests with Bonferroni correction revealed a significant difference between the *single-probe* (*M* = 0.846) and *item recognition* (*M* = 0.781) tests (*p* < 0.01).

#### Parameter estimates

The ROCs were further analyzed by fitting them to a mixture signal detection model to estimate the probability of recollecting whether a change occurred (R_Change_), whether a repetition occurred (R_Repetition_), and whether familiarity contributed to performance (*d′*). When examining the parameter estimates for recollection (see Fig. 3C), a repeated-measures ANOVA revealed a significant interaction between test type and the type of recollection. *F*(2,44) = 14.88 *p* < 0.001, indicating that the probability of recollecting repetitions and changes depended on the type of test. Subsequent Bonferroni-corrected *t*-tests revealed that this interaction arose because recollecting a repetition (R_Repetition_) occurred more often in the *item recognition* test than in the *complex-probe* test, *t*(22) = 3.531, *p* < 0.01, whereas recollecting a change (R_Change_) occurred more often in the *complex-probe* test than in *item recognition* test, *t*(22) = 3.866, *p* < 0.01. In addition, recollecting a repetition (R_Repetition_) occurred more often in the *single-probe* test than in the *complex-probe* test, *t*(22) = 2.598, *p* < 0.05, whereas recollecting a change (R_Change_) occurred more often in the *single-probe* test than in the *item recognition* test, *t*(22) = 2.953, *p* < 0.05. When comparing within test-type, R_Change_ occurred more frequently than R_Repetition_ in the *complex-probe* test, *t*(22) = 6.383, *p* < 0.001, and R_Change_ also occurred more often than R_Repetition_ in the *single-probe* test, *t*(22) = 2.328, *p* < 0.05. In contrast, R_Repetition_ was numerically higher than R_Change_ in the *item recognition* test but the difference was not significant, *t*(22) = 1.014, *p* > 0.05.

An examination of the familiarity estimates (*d′*) indicated that familiarity was equally useful in all three tests (see Fig. 3D). That is, a one-way repeated-measures ANOVA revealed a nonsignificant difference between the mean scores of familiarity across tests, *F*(2,44) = 0.578, *p* > 0.05.

## Experiment 2: Blocked testing

### Methods

#### Participants, stimuli, and procedure

Thirty-three participants were recruited from the undergraduate population at the University of California, Davis. 4 participants were excluded due to a lack of range in responses to evaluate ROC shape (i.e., having less than 3 ROC points), resulting in a final sample of 29 participants. Age ranged from eighteen to thirty-one years. The materials used were identical to those in Experiment 1, but the three tests were presented in a blocked format. Participants completed one type of test at a time, with a two-minute break between blocks. The order of tests was counterbalanced to minimize order effects. During pilot testing of Experiment 2, performance reached ceiling for many participants, prompting a reduction in presentation time from 500 to 300 ms to increase test difficulty. The distribution of confidence responses for each blocked test can be found in Supplementary Table 2.

### Results

#### ROCs shape

**Fig. 4:**
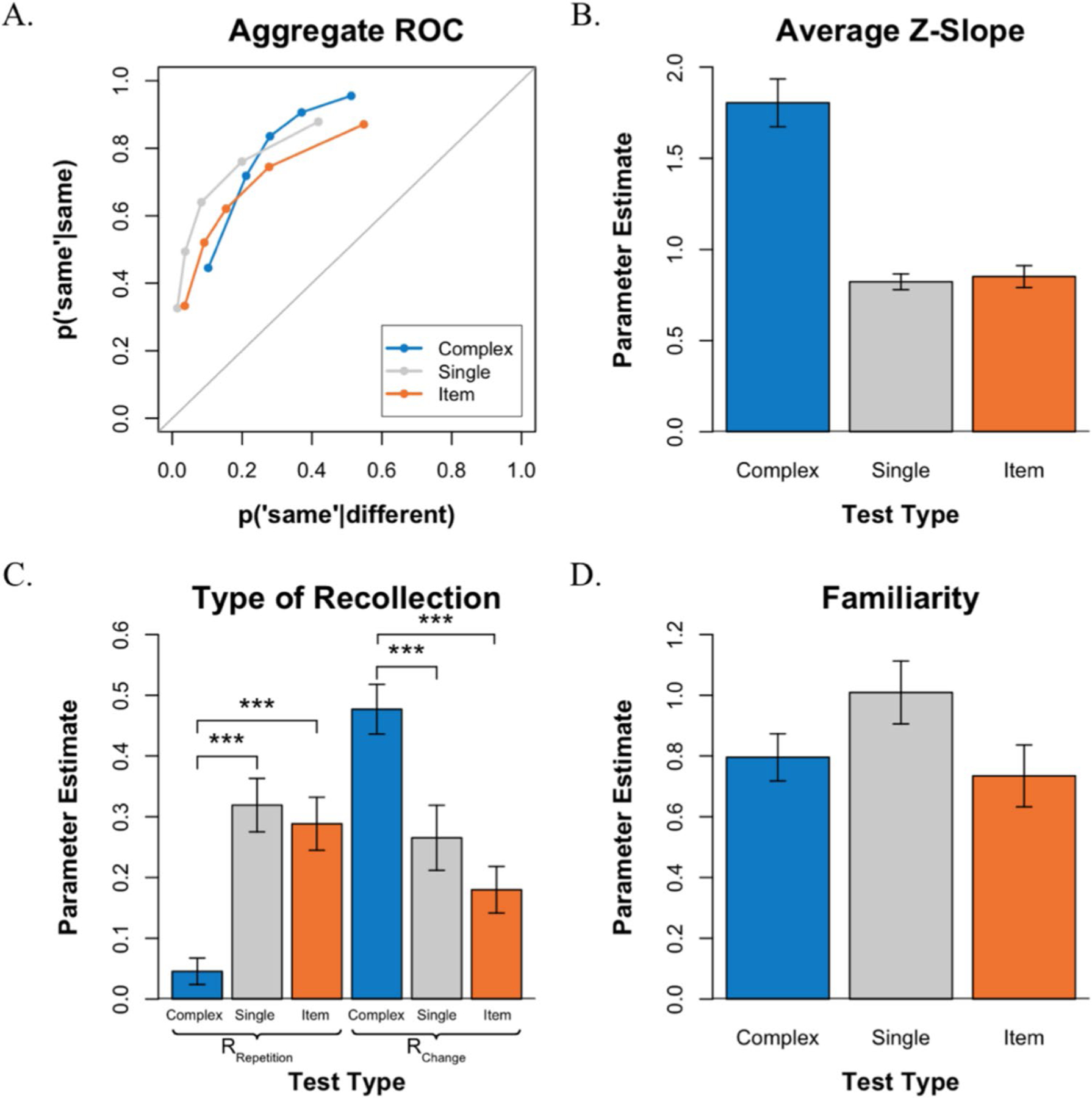
VWM performance for each test in Experiment 2. **A.** Average ROCs for the three tests. **B.** Average *z*-slope estimates across tests. **C.** Estimates of recollection type (R_Repetition_ vs. R_Change_) across tests. **D.** Estimates of familiarity across tests. ***p < 0.001

Consistent with Experiment 1, the ROCs exhibit an inverted U-shape, and the degree of asymmetry varied across test conditions (see Fig. 4A). An examination of the average ROC *z*-slopes (see Fig. 4B) revealed that the slopes varied significantly across conditions, *F*(2,56) = 37.25, *p* < 0.001. As was observed in Experiment 1, in the *complex-probe* test, the mean *z-*slope was 1.805, which was significantly greater than 1.0, *t*(28) = 6.123, *p* < 0.001, and in the *item recognition* test, the mean *z*-slope was 0.851 which was significantly less than 1.0, *t*(28) = − 2.46, *p* < 0.05. However, in contrast to Experiment 1, in the *single-probe* test, the ROC was not symmetrical with a slope of 1.0 but rather exhibited a shallow slope that was significantly less than 1.0 (*M* = 0.822), *t*(28) = − 4.12, *p* < 0.001. Finally, overall performance in the three tests was roughly comparable, as evidenced by the overlapping ROCs, and an examination of the AUC indicated that overall discriminability varied across tasks, *F*(2,56) = 7.736, *p* < 0.01. Further comparisons using paired *t* tests with Bonferroni correction revealed the same significant difference as in Experiment 1 between the *single-probe* (*M* = 0.842) and *item recognition* (*M* = 0.787) tests (*p* < 0.01).

#### Parameter estimates

The ROCs were examined further by fitting them to the mixture signal detection model to estimate R_Change_, R_Repetition_, and *d′* (see Fig. 4C–D). Consistent with Experiment 1, a repeated-measures ANOVA revealed a significant interaction between test type and the type of recollection, *F*(2,56) = 28.96, *p* < 0.001, indicating that the probability of recollecting repetitions and recollecting changes depended on test type. Subsequent Bonferroni-corrected *t*-tests revealed that this interaction arose, again, because recollecting a repetition (R_Repetition_) occurred more often in the *item recognition* test than in the *complex-probe* test, *t*(28) = 5.195, *p* < 0.001, whereas recollecting a change (R_Change_) occurred more often in the *complex-probe* test than in *item recognition* test, *t*(28) = 5.744, *p* < 0.001. Additional *t*-tests indicated that recollecting a repetition (R_Repetition_) occurred less often in the *complex-probe* test compared with the *single-probe, t*(28) = − 5.896, *p* < 0.001, whereas recollecting a change (R_Change_) occurred more often in the *complex-probe* test compared with the *single-probe* test, *t*(28) = 4.367, *p* < 0.001. When comparing within test-type, R_Change_ was greater than R_Repetition_ in the *complex-probe* test, *t*(28) = 9.844, *p* < 0.001, while R_Repetition_ was greater than R_Change_ in the *item recognition* test, *t*(28) = 2.122, *p* < 0.05. In contrast, R_Repetition_ was numerically higher than R_Change_ in the *single-probe* test but the difference was not significant, *t*(28) = 0.866, *p* > 0.05.

Thus, the parameter estimates of recollection in the current experiment replicated the pattern of results observed in Experiment 1, with one minor difference: recollection estimates in the *single-probe* test varied slightly. Numerically, the estimates of R_Repetition_ appeared slightly higher, while the estimate of R_Change_ appeared slightly lower in Experiment 2. However, statistical comparisons across experiments revealed that these apparent differences, as well as comparisons of all other parameter estimates, were not statistically significant. We consider these findings further in the discussion.

An ANOVA examining the familiarity *d′* estimates (see Fig. 4D) indicated that familiarity was not equally useful in all three tests, *F*(2,56) = 4.146, *p* < 0.05. However, individual *t*-tests with Bonferroni correction revealed no significant differences in estimates of familiarity between test types (*p* > 0.05).

## Discussion

The current study examined the role of change detection and repetition detection in VWM. Although prior work had suggested that the role of these processes may depend on the type of memory test (Aly & Yonelinas, 2012; Rhodes et al., 2018; Rouder et al., 2008, 2011; Yonelinas, 2024), the current study is the first to directly test these ideas. Experiment 1 examined working memory in three types of tests (i.e., *complex-probe*, *single-probe*, and *item recognition*) under conditions where materials were held constant, but the type of test varied randomly from trial to trial, such that during encoding, subjects did not know which type of test they would receive. As predicted, in the *complex-probe* test, the slope of the observed ROC was steep, whereas in the *item recognition* test, the slope was shallow, and in the *single-probe* condition, the slope was intermediate. The shallow slope in the *item recognition* test shows that repetitions led to disproportionately more accurate high confidence “same” responses (i.e., the task relied heavily on repetition detection), whereas the steep slope in the *complex-probe* test shows that changes led to disproportionately more accurate high confidence “different” responses (i.e., the task relied heavily on change detection), and the symmetrical ROC in the *single-probe* test shows that repetitions and changes led to similar levels of accurate high confidence responses (i.e., the task relied similarly on repetition detection and change detection).

To further quantify these effects, the ROCs were fit to a mixture signal detection model, which confirmed that the likelihood of recollecting changes and recollecting repetitions varied significantly across tests, such that recollection of changes occurred more in the *complex-probe* than in the *item recognition* test, whereas the recollection of repetitions occurred more in the *item recognition* than in the *complex-probe* test. In contrast, in the *single-probe* test, recollection of repetitions and changes played comparable roles in performance.

Experiment 2 was similar to Experiment 1, except that the test conditions were blocked rather than being presented in random order, allowing subjects to adopt different encoding strategies for each of the test types. The ROCs and parameter estimates obtained in Experiment 2 closely mirrored those from Experiment 1, indicating that the *complex-probe* test preferentially relied on change detection, whereas the *item recognition* test preferentially relied on repetition detection.

The present results are consistent with prior studies that suggested that ROC symmetry depended critically on the type of memory test (Aly & Yonelinas, 2012; Rhodes et al., 2018; Rouder et al., 2008, 2011; Yonelinas, 2024), but those earlier results were ambiguous as there were differences in materials and test procedures that could have explained the observed results. Here, for the first time, we directly contrasted the ROCs in these three tests while controlling for materials and other procedural differences, allowing us to conclude that test type is responsible for determining ROC asymmetry. In addition, the results indicated that the observed differences across tests were due to increases in the likelihood that changes and repetitions were recollected. That is, the increase in accurate high-confidence change detections in the *complex-probe* test reflected an increase in the likelihood that subjects recollected a change (i.e., “It was blue, not yellow”), whereas the increase in accurate high-confidence repetition detections in the *item recognition* test reflected an increase in the likelihood that subjects recollected a repetition (i.e., “It was blue, and this did not change”). Parameter estimates of familiarity did not change across test types, suggesting that familiarity was equally useful across these test conditions.

One unexpected result was that in the *single-probe* test, the ROC was symmetrical when the test trials were randomly intermixed (Experiment 1), whereas it was asymmetrical with a shallow slope when the test trials were blocked (Experiment 2). Parameter estimates suggest that the shallower slope in the *single-probe* test from Experiment 2 reflects a greater reliance on the recollection of repetitions compared with changes under blocked testing conditions. This may indicate a general tendency for subjects to orient more to repetitions than to changes when confronted with repeated *single-probe* trials. However, we emphasize that these differences in parameter estimates across experiments were not statistically significant, so future studies examining whether subjects can strategically orient toward detecting changes versus repetitions will be important in testing this hypothesis. In addition, a majority of previous experiments using *single-probe* tests—all of which used blocked testing procedures—produced symmetrical ROCs (Robinson et al., 2020; Xie & Zhang, 2017b). Notably, however, one of four experiments in Xie and Zhang (2017a) reported a steep ROC slope. Our finding of a shallow slope, alongside this earlier result, suggests that different test conditions may lead to a subtle shift in the relative contribution of change detection and repetition detection in *single-probe* tests.

Another potentially informative difference between the two experiments emerged in the *item recognition* tests. That is, although estimates of recollecting a repetition were generally greater than recollection of changes in *item recognition*, the difference was only statistically significant in Experiment 2. A reviewer pointed out an interesting possibility that the *item recognition* test may be viewed as a partial form of the *single-probe* test, as both require evaluating a single probe against memory for studied items and place similar demands on recollection. This similarity could help explain the comparable performance observed between the two tests across experiments, particularly in Experiment 2. The results indicate that although recollection of repetitions occurred more frequently than recollection of changes in tests of *item recognition*, subjects are also able to use recollection to identify when a changed item was not studied. The finding that *item recognition* relies heavily on the recollection of repetitions is consistent with previous studies of working memory that reported *z*-slopes of less than 1.0 (Robinson et al., 2020; Yonelinas & Jacoby, 1997, as reported in Yonelinas, 2024), and is also consistent with studies of episodic memory tests of *item recognition,* in which *z*-slopes are almost always less than 1.0 (for review, see Yonelinas & Parks, 2007). However, one exception to this pattern is a working memory study of *item recognition* for Gabor patches, which reported *z-*slopes that were greater than 1.0 (Yotsumoto et al., 2008). Why the results of that study differed is unknown, but it was unique in several ways (e.g., it used a set of highly similar Gabor patches, sequential three-item study lists, and a continuous visual rating procedure rather than a numerical confidence scale). If the current approach is correct, it would suggest that under those conditions, subjects were utilizing change detection more so than repetition detection. However, future studies will be needed to directly test this account and to determine which of the unique procedural aspects of that study led to the observed effects.

### Implications

The current results have several theoretical and practical implications. First, they indicate that change detection and repetition detection can be functionally dissociated, in the sense that the relative roles of these two processes differed quite dramatically across tests. For example, there were significant interactions observed between the ROC slope measures and test type, as well as crossover interactions in the extent to which R_Repetition_ and R_Change_ contributed to memory in the *complex-probe* and *item recognition* tests—R_Repetition_ was greater in the *item recognition* than in the *complex-probe* test, whereas R_Change_ showed the opposite pattern, being greater in the *complex-probe* than in the *item recognition* test. These dissociations suggest that VWM reflects the combined effects of two distinct memory processes or components.

An important open question that remains to be explored is whether these two forms of working memory are differentially influenced by other experimental manipulations. For example, it is possible that the roles of change and repetition detection may differ substantially when using more complex, real-world materials. Additionally, other factors—such as changes in the number of items in the study list, as well as variations in the encoding or delay conditions—may also impact the processes that contribute to working memory performance.

Considering the distinction between change and repetition detection could also significantly impact our understanding of working memory across various populations. That is, the present results raise the question of whether certain individuals, due to neurological differences, would struggle more with detecting changes in their environment while retaining the ability to detect repetitions—or vice versa. For example, healthy aging has been associated with a reduction in working memory, but it remains unclear whether aging affects either change detection or repetition detection more. Additionally, the possibility of distinct subpopulations exhibiting unique patterns of deficits in these two processes has yet to be explored.

In addition, the behavioral dissociations that were observed in the current study raise the question of whether change detection and repetition detection are neurally distinct. That is, could the neural process of detecting a visual change be distinct from the process of detecting a repetition? Although there is an extensive body of literature using methods such as ERPs and fMRI to examine the neural basis of VWM (e.g., Beck et al., 2001; Huettel et al., 2001; Mayer et al., 2007; Song & Jiang, 2006), we are not aware of any that have directly contrasted change detection and repetition detection. However, in a recent fMRI study of episodic memory (Bowman & Dennis, 2016), different regions were related to trials in which subjects used memory to detect repetitions, compared with those used to detect changes. Specifically, they found that recollection rejection (i.e., change detection) was supported by a lateral frontoparietal network, as well as the orbitofrontal cortex (OFC), while target recollection (i.e., repetition detection) was supported by activity in the bilateral hippocampus, medial prefrontal regions, and the dorsal anterior cingulate. Whether change detection and repetition detection also rely on distinct networks in working memory should be explored.

The current results indicate that while recollection was highly sensitive to test type, familiarity remained consistent across all three tests. Notably, we did not have any strong a priori predictions about how familiarity would behave in these studies. However, prior confidence ROC studies have demonstrated curvilinear ROCs across these paradigms, suggesting that familiarity contributes to discriminating between “same” and “different” items in all of them. Our findings of curved ROCs align with previous work, reinforcing the role of familiarity in these tests.

### Alternative models

The current results were fit to a mixture signal detection model which revealed that observed differences in the shapes of the memory strength distributions for “same” and “different” items across the three memory tests reflected the varying contributions of recollecting changes versus recollecting repetitions. In contrast, familiarity-based responses appeared to contribute equally across all test types. How does this model relate to other working memory models, and might they lead to different conclusions?

In general, the current results rule against single-component models of working memory such as single-parameter threshold models that are often used to measure memory capacity, and the standard signal detection model that is often used to measure memory sensitivity (*d′*). The problems with these models have been discussed in many previous papers (Kellen & Klauer, 2015; Rouder et al., 2011; Yonelinas & Parks, 2007), and we will not reiterate them here, except to point out that without additional assumptions, these models cannot account for the dissociations in the ROC shapes that were observed across these different memory tests (i.e., the signal detection model can only produce symmetrical ROCs, and the threshold models can only produce linear ROCs).

One model that can account for the current results and produces ROCs that closely resemble those generated by the mixture model, is the unequal variance signal detection model (Hautus et al., 2021; Yonelinas & Parks, 2007). In fact, when both recollection and familiarity contribute to performance, as in the current experiments, the two models can produce ROCs that are virtually identical, and thus direct statistical comparisons are not particularly useful (Yonelinas & Parks, 2007). Although there are a number of findings that present challenges for the unequal-variance model in studies of episodic memory (e.g., Decarlo, 2002; Dobbins, 2023; Yonelinas & Parks, 2007) and working memory (Aly et. al., 2012; Yonelinas 2024), in the current study, the model leads to conclusions that are largely consistent with those based on the mixture model. That is, the unequal variance model has a familiarity strength component (i.e., *d′*) like that of the mixture model, but rather than assuming there is an additional recollection process contributing to high-confidence responses, it assumes that there is a separate process or mechanism that increases or decreases the relative variance of the “same” or “different” familiarity strength signals. One interpretation of this “extra variance” component is that it reflects a recollection process that can contribute either to the “same” or the “different” items. Under this interpretation, the conclusions would be essentially identical to those of the mixture model: recollection of changes leads to the steep ROC slope in the *complex-probe* test, recollection of repetitions leads to the shallow slope in the *item recognition* test, and recollection of both changes and repetitions in the *single-probe* test leads to more symmetrical ROCs. However, other interpretations of the “extra variance” component are also possible. For example, in episodic memory studies, it has been argued that old item distributions may have greater variance than new items because there is encoding variability that increases the variance of old items but not new items (Wixted, 2007). Although studies have not yet found any evidence in support of this possibility in episodic memory (Koen et al., 2013; Koen & Yonelinas, 2010, 2013; Spanton & Berry, 2020; Starns et al., 2012), whether this approach may fare better in tests of working memory is not yet known. In addition, in global matching models of episodic memory (Gillund & Shiffrin, 1984; Hintzman, 1988; Shiffrin & Steyvers, 1997), greater target variability can emerge from complex interactions arising from noisy encoding and nonlinear retrieval processes. Again, however, whether such approaches would be able to account for the observed dissociations observed in working memory ROCs is unknown.

The current proposal that working memory relies on recollection and familiarity is broadly consistent with some aspects of other multiple-component models of working memory. For example, Cowan has suggested that working memory reflects the contents of a limited-capacity focus of attention, as well as the activation of long-term memory representations (Cowan et al., 2021). Similarly, Oberauer has argued that working memory reflects direct access to a context frame that temporarily links studied items together, as well as activation of long-term memory representations (Lin & Oberauer, 2022; Oberauer, 2013; Oberauer & Lin 2017). In contrast, other models have included signal detection-based memory signals, plus some form of random guessing (Luck & Vogel, 2013) or random noise (Bays et al., 2009; Schurgin et al., 2020). It seems reasonable to assume that recollection may reflect the contents of a limited-capacity focus of attention (Cowan et al., 2021), or the direct access to a context frame (Oberauer & Lin, 2017), and that when these processes are successful, they would support both high-confidence “same” responses and high-confidence “different” responses. In addition, activation of long-term memory representations may give rise to a familiarity signal that is consistent with a signal detection-like familiarity process, which may be influenced by guessing or noise. Thus, the familiarity and recollection processes underlying the current model do seem broadly consistent with at least some of the constructs proposed in other models. However, these models have not yet been applied to working memory ROC results, and thus, we do not know if they are consistent with the existing literature or how they relate to the processes identified in the current studies.

Another modeling approach that may also be consistent with the current results is the bump-attractor model (e.g., Compte et al., 2000; Wei et al., 2012), in which performance relies on multiple cell assemblies, each of which holds qualitative information about a separate study item (e.g., “The upper right square is green”). Although it has not been directly applied to ROC results, these signals may be mixed together to produce at least some of the ROCs that were observed. For example, in the *complex-probe* test, each of the five test items could be used as a memory cue and so could produce five separate memory signals. For the “same” trials, this would lead to five “match” signals (i.e., all test cues matched what was stored in memory), whereas for a “different” trial, it would lead to four “match” signals and one “mismatch” signal. In this way, there would be more variance in the memory strength signals in the “different” trials than in the “same” trials, and so this could produce a steep ROC, as was observed in the current experiments. In contrast, in an *item recognition* test, the test item could be compared with the contents of each of the five studied items being held in working memory, and so for a “same” trial, this would lead to one “match” and four “mismatch” signals, whereas a “different” trial would lead to five “mismatch” signals. Thus, the “same” trials would have more memory variability than the “different” trials, potentially leading to a shallow ROC slope, as was also observed in the current experiments. However, in a *single-probe* test, the “same” trials would lead to one “match” and four “mismatch” signals, whereas the “different” trials would lead to five “mismatch” signals, and so the “same” trials would have greater variance, leading to a shallow slope. These results are inconsistent with the results of Experiment 1, in which the ROCs were found to be symmetrical (and with most previous *single-probe* studies), but they are consistent with Experiment 2, in which the ROCs exhibited a shallow slope. So, there is at least some support for the utility of this approach.

### Which visual working memory test is the easiest?

While it may seem like this question should have a straightforward answer, our results suggest otherwise. For example, one might intuitively expect the *item recognition* test to be relatively easy, as it only requires memory for item information (e.g., “Was there a blue item in the array”), compared with the *single-probe* test, which requires participants to remember both the item and its location (e.g., “Was the item in this location blue?”). However, our observed results indicate that overall discriminability was actually lower in the *item recognition* test. In fact, a close examination of the ROCs shows that for the highest-confidence “same” responses (i.e., the left side of the ROC), performance was essentially identical in the two tests (*item recognition* and *single-probe* curves intersect at this point). Yet, when lower-confidence responses are included (i.e., the points more to the right side of the ROC), an accuracy advantage for the *single-probe* test emerges (i.e., the ROC is now higher for the *single-probe* test than for the *item recognition* test). These results can be explained by considering the model parameter estimates, which indicated that the probability of recollecting a repetition was equally likely in the *item recognition* and *single-probe* tests. However, because the probability of recollecting a change was much greater in the *single-probe* test than in the *item recognition* test, performance on the right side of the ROC—which is influenced by change detection—shows a *single-probe* advantage. Together, these results suggest that different working memory tests do not simply differ in overall difficulty or response criterion placement, but rather in the extent to which they rely on different underlying memory processes.

## Conclusion

To examine the roles of change detection and repetition detection in VWM, we analyzed three working memory tests that are expected to differentially rely on these processes (*single-probe*, *complex-probe*, and *item recognition* tests). By collecting confidence-rated “same/different” responses and analyzing receiver operating characteristics (ROCs), we evaluated memory strength distributions for changed and repeated trials and used a mixture signal detection model to estimate change and repetition detection parameters. As expected, the *complex-probe* test showed more high-confidence memory for “different” trials, given subjects need only recollect a single changed item to confidently accept the trial as having changed. In contrast, the *item recognition* test showed more high-confidence memory for repetitions, as subjects need only recollect one item as having been studied to confidently accept the trial as being repeated. The *single-probe* test showed similar confident memory for both types of trials, as recollection should be roughly equally useful in detecting a change or a repetition. These results demonstrate that change detection and repetition detection are functionally dissociable forms of working memory. We are optimistic that future studies examining working memory ROCs will prove valuable in understanding the conditions that influence these processes, identifying which processes are disrupted in vulnerable populations, and uncovering their underlying neural mechanisms.

## Supplementary Material

Supp

**Supplementary Information** The online version contains supplementary material available at https://doi.org/10.3758/s13421-025-01749-2.

## Figures and Tables

**Table 1:** 8-Bit RGB [0:255] values of stimuli

Color	RGB value		
	R	G	B
Green	0	255	51
Yellow	255	255	0
Red	255	51	0
Pink	255	0	153
Purple	153	0	255
Blue	0	51	255
Cyan	0	255	255
Brown	150	75	0
Orange	255	140	0

## Data Availability

The datasets generated during and/or analyzed during the current study are available on OSF.
