# Supplementary material for: Change detection and repetition detection reflect functionally distinct forms of visual working memory
Source: Mem Cognit. Author manuscript; Available in PMC 2025 Aug 4. (PMC12321045; doi:10.3758/s13421-025-01749-2)
Supplement: Supplementary file 1 [file NIHMS2093865-supplement-Supp.docx]

**Supplementary Table 1*****.*** *Number of responses at each level of confidence for Experiment 1.*

| Task Type | 1 | 2 | 3 | 4 | 5 | 6 |
| --- | --- | --- | --- | --- | --- | --- |
| Complex New | 604 | 219 | 110 | 102 | 171 | 174 |
| Complex Old | 37 | 49 | 62 | 129 | 405 | 698 |
| Single New | 725 | 366 | 154 | 69 | 45 | 21 |
| Single Old | 114 | 213 | 198 | 230 | 306 | 319 |
| Item New | 634 | 387 | 194 | 86 | 57 | 22 |
| Item Old | 193 | 217 | 200 | 180 | 247 | 343 |

 

**Supplementary Table 2*****.*** *Number of responses at each level of confidence for Experiment 2.*

 

| Task Type | 1 | 2 | 3 | 4 | 5 | 6 |
| --- | --- | --- | --- | --- | --- | --- |
| Complex New | 849 | 246 | 158 | 119 | 189 | 179 |
| Complex Old | 78 | 85 | 123 | 204 | 475 | 775 |
| Single New | 1012 | 381 | 202 | 81 | 39 | 25 |
| Single Old | 211 | 206 | 209 | 254 | 293 | 567 |
| Item New | 786 | 471 | 215 | 109 | 97 | 62 |
| Item Old | 225 | 219 | 216 | 174 | 327 | 579 |
